# A case series of continuous paravertebral block in minimally invasive cardiac surgery

**DOI:** 10.1186/s40981-017-0119-0

**Published:** 2017-08-29

**Authors:** Shintaro Tahara, Akito Inoue, Hajime Sakamoto, Yasuaki Tatara, Kayoko Masuda, Yoichiro Hattori, Yusaku Nozumi, Mitsumasa Miyagi, Surakshya Sigdel

**Affiliations:** 1 Department of Anesthesia, Akashi Medical Center, 743-33 Yagi, Ookubo-cho, Akashi, Hyogo 674-0063 Japan; 2 Department of Anesthesia, Ohnishi Neurological Center, 1661-1 Eigashima Ookubo-cho, Akashi, Hyogo 674-0064 Japan

**Keywords:** Paravertebral block, Minimally invasive cardiac surgery, Perioperative analgesia

## Abstract

**Background:**

Minimally invasive cardiac surgery (MICS), via minithoracotomy, is thought to be a fast track to extubation and recovery after surgery. For this, good coverage analgesia is essential. Epidural anesthesia, a standard technique for thoracic surgery, has high risk of complications, such as epidural abscess and spinal hematoma in open-heart surgery. Based on the hypothesis that continuous paravertebral block (CPVB), a less invasive regional anesthetic technique, is safe and effective in open-heart surgery, we applied CPVB to MICS with thoracotomy.

**Findings:**

To assess whether CPVB could be used in open-heart surgery with fewer potential complications, we investigated our medical records of the 87 adult patients who underwent MICS at Akashi Medical Center, Hyogo, Japan, between March 2009 and May 2016. We collected data of CPVB-related complications, postextubation respiratory failure, duration of intubation, and other analgesic use from hospital clinical records. We observed no severe CPVB-related complications, such as hematoma, neuropathy, or abscess. PT-INR longer than 1.1 was associated with CPVB-related minor bleeding. Forty-three patients (47.4%) were extubated within 1 h after surgery, and there were no postextubation respiratory failures in any patients.

**Conclusions:**

We observed no cases of severe CPVB-related complications or postextubation respiratory failure in any of our patients who underwent MICS. Preoperative prolongation of PT-INR was associated with CPVB-related minor bleeding.

## Background

Minimally invasive cardiac surgery (MICS) is a surgical procedure with alternate minimal access incisions, on-pump beating heart techniques, off-pump valve repair devices, robotics, and/or transcatheter devices [1]. MICS with lateral thoracotomy is used in various cardiac surgeries, such as mitral valve repair (MVR), aortic valve repair (AVR), and minimally invasive direct coronary artery bypass (MIDCAB). MICS with lateral thoracotomy is expected to reduce surgical bleeding, duration of intubation, intensive care unit (ICU) stay and perioperative mortality [1–3]. However, if poorly managed, lateral thoracotomy is associated with postoperative pain and leads to complications, such as postoperative pneumonia [4–9]. Epidural anesthesia (EA) is the current gold standard for post-thoracic surgery analgesia. However, it is contraindicated for patients requiring anticoagulant or heparinization and leads to possible complications, such as epidural hematoma and neuropathy [10]. With increasing interest in regional block techniques, ultrasound-guided continuous paravertebral block (CPVB) has been shown to be effective and safe for postoperative analgesia after thoracic surgery [11–15]. In this report, we retrospectively examined whether CPVB was safe and effective in open-heart surgery.

## Methods

This is a case series without clinical intervention. This study was approved by the ethical committee of Akashi Medical Center on 17 October 2016 (approval number: 28-14). We collected demographic and clinical data of 87 adult patients who had undergone elective MICS with lateral thoracotomy between March 2009 and May 2016. All patients were well informed on the procedure, and consent for continuous paravertebral block (CPVB) was obtained prior to surgery. We retrospectively assessed CPVB-related complications, postextubation respiratory failure, duration of intubation after surgery, perioperative fentanyl consumption, and other analgesic use.

### Anesthetic and analgesic protocol

In all patients, sedation was performed with midazolam (1 mg) and fentanyl citrate (100 μg) followed by induction with thiamylal sodium (3–4 mg/kg), esmolol hydrochloride (1 mg/kg), and vecuronium bromide (0.1 mg/kg) immediately after the radial artery was cannulated. Anesthesia was maintained with sevoflurane 1.5% and intermittent fentanyl and vecuronium injections at the discretion of anesthesiologist. After induction, intubation was performed with a single-lumen tube (Parker Flex-Tip™ PFHV, Japan Medicalnext, Osaka, Japan) and bronchial blocker tube (COOPDECH Endobronchial Blocker Tube, Daiken Medical, Osaka, Japan) or a double-lumen tube (Mallinckrodt™ Endobronchial Tube, Covidien Japan, Tokyo, Japan) for one-lung ventilation. A pulmonary artery catheter (Swan-Ganz CCOmbo V, Edwards Lifescience Japan, Tokyo, Japan) was inserted via the left internal jugular vein. An 18-gauge intravenous line was inserted via the right internal jugular vein in order to facilitate placement of the cannula to drain venous flow from the superior vena cava during cardiopulmonary bypass. For the initiation of cardiopulmonary bypass, 300 units/kg of unfractionated heparin was administered to achieve activated coagulation time of more than 400 s. Routine monitoring included continuous arterial, central venous, and pulmonary artery pressure, pulse oximetry, five-lead electrocardiogram, regional saturation of oxygen of brain (INVOS OXIMETER, Covidien Japan, Tokyo, Japan), bladder temperature, end-tidal carbon dioxide concentration, airway pressure, and transesophageal echocardiography (EPIQ 7, Philips Electronics Japan, Tokyo, Japan).

**Fig. 1:**
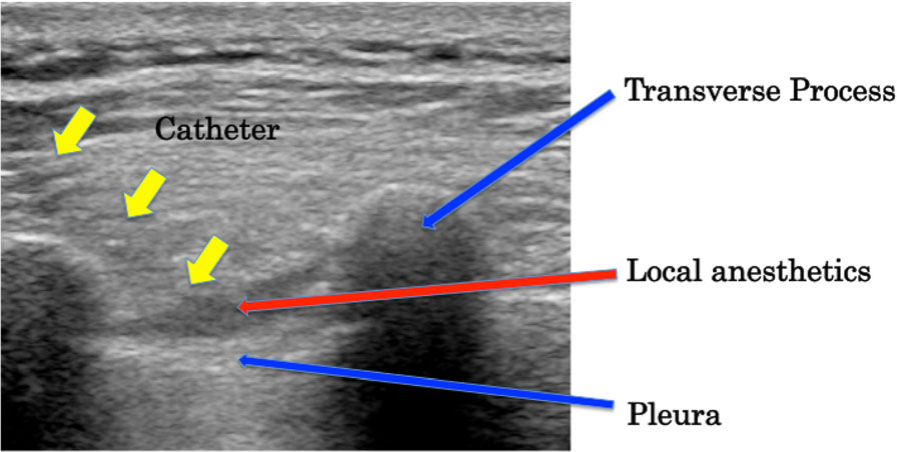
Ultrasound-guided paravertebral block. We confirmed that local anesthetic infused via the catheter spread in paravertebral space

Ultrasound-guided CPVB was performed after induction of anesthesia in the lateral decubitus position. We confirmed that no antiplatelet or anticoagulant was administered preoperatively. An 18-gauge Tuohy’s needle was placed at T5/6 or T6/7 paravertebral spaces using an in-plane approach followed by a catheter (Perifix custom kit, B Braun Aesculap Japan, Tokyo, Japan). We performed bolus infusion of 40–60 mL ropivacaine 0.2% followed by continuous infusion of 4–6 mL/h of ropivacaine 0.2%. We confirmed by ultrasound that local anesthetic infused via the catheter spread in paravertebral space successfully in all patients (Fig. 1). Continuous infusion of ropivacaine and 3 mL bolus with a 60-min lockout interval was postoperatively maintained for 48–72 h under patient-controlled analgesia (PCA) (Coopdech balloonjecter 300, Daiken Medical, Osaka, Japan).

### Postoperative assessment

We postoperatively assessed CPVB-related complications every 2 hours: nausea and vomiting, leakage of local analgesics, bleeding, neuropathy, and abscess. If required, we gave patients a postoperative analgesic regimen consisting of intravenous pentazocine hydrochloride (15 mg), intravenous buprenorphine hydrochloride (0.2 mg), and oral loxoprofen sodium hydrate (60 mg). Complication and analgesic use was recorded by nurses.

### Outcomes

CPVB-related postoperative complications included leakage of local analgesic and bleeding from the puncture point, neuropathy, abscesses, nausea and vomiting.

Postextubation respiratory failure was defined as the presence and persistence of any of the following: postoperative pneumonia, respiratory acidosis (pH < 7.35 with PaCO_2_ > 45 mmHg), hypoxemia (SpO_2_ < 90% or PaO_2_ < 60 mmHg with FiO_2_ ≥ 0.5), tachypnea > 35 breaths/min, respiratory muscle fatigue, and/or low level of consciousness [6]. Postoperative pneumonia was defined as the presence and persistence of signs of respiratory infection, such as fever, purulent sputum, leukocytosis, increased level of blood C-reactive protein, and worsening oxygenation with a new radiographic infiltrate [6].

### Statistical analysis

Continuous variables are presented as means and standard deviations. Chi-square test was used to estimate relevance of international normalized ratio of prothrombin time (PT-INR)- and CPVB-related bleeding. *P* value of < 0.05 was considered statistically significant.

## Findings

Table 1 shows patient characteristics. As shown in Table 2, minor bleeding or leakage of local anesthetics, nausea, and vomiting were observed in 15 (17.2%) patients. PT-INR longer than 1.1 was associated with CPVB-related bleeding (Table 3). All nine patients with minor bleeding had been administered postoperative intravenous heparin. We observed no other complications, for example, severe hematoma, neuropathy, or abscess. No patients had postextubation respiratory failure or reintubation. Forty-three patients (47.4%) were extubated within 1 hour after surgery. The number of other analgesic use was 0.9 ± 0.9, and no patients required postoperative continuous intravenous infusion of opioids during CPVB. The intraoperative fentanyl consumption was 366.7 ± 236.7 μg.

**Table 1:** Patient characteristics (*n* = 87)

Characteristics	
Age (years)	65 ± 12
Sex (male)	44 (51%)
Height (cm)	161 ± 10
Body weight (kg)	55 ± 12
Surgery time (min)	241 ± 53
Anesthesia time (min)	361 ± 57
Surgical procedure	
MVR	73 (84%)
AVR	2 (2%)
VSD or ASD closure	7 (8%)
MIDCAB	1 (1%)
Resection of myxoma	4 (5%)
Time from CPVB to heparin (min)	57 ± 18
Preoperative platelet count (×10^4^/μL)	22 ± 7
Preoperative PT-INR	1 ± 0.3
Duration of CPVB (days)	2 ± 1
Postoperative anticoagulant and antiplatelet	
Intravenous heparin	76 (87%)
Aspirin or other antiplatelet	0 (0%)

Values are mean ± SD or numbers (%)

*CPVB* continuous paravertebral block, *MVR* mitral valve repair, *AVR* aortic valve repair, *VSD* ventricular septal defect, *ASD* atrial septal defect, *MIDCAB* minimally invasive direct coronary artery bypass, *PT-INR* international normalized ratio of prothrombin time

**Table 2:** Outcomes (*n* = 87)

Results of outcomes	
CPVB-related complications	15 (17.2%)
Nausea and vomiting	1 (1.1%)
Leakage of local analgesic	5 (5.8%)
CPVB-related minor bleeding	9 (10.3%)
CPVB-related neuropathy	0 (0%)
CPVB-related abscess	0 (0%)
Postextubation respiratory failure	0 (0%)
Duration of intubation after surgery (hours)	4.8 ± 5.9
< 1 h	43 (49.4%)
≥ 1 h, < 12 h	28 (32.2%)
≥ 12 h	16 (18.4%)
Fentanyl consumption (μg)	366.7 ± 236.7
Number of other analgesic use	0.9 ± 0.9
= 0	11 (12.6%)
= 1	53 (60.9%)
≥ 2	23 (26.4%)

Values are mean ± SD or numbers (%)

*ICU* intensive care unit, *CPVB* continuous paravertebral block

**Table 3:** Relationship between PT-INR- and CPVB-related bleeding

PT-INR	Patients with CPVB-related bleeding	Patients without CPVB-related bleeding	OR (95%CI)	*P* value
	*n* = 9	*n* = 78		
≧ 1.0	9	51	9.53 (0.53–170.74)	0.061^a^
< 1.0	0	27		
≧ 1.1	6	22	5.09 (1.17–22.17)	0.0193^a^
< 1.1	3	56		
≧ 1.2	5	7	12.68 (2.75–58.38)	0.0001^a^
< 1.2	4	71		
≧ 1.3	3	4	9.25 (1.67–51.28)	0.0033^a^
< 1.3	6	74		

Relationship between PT-INR- and CPVB-related bleeding

*PT-INR* international normalized ratio of prothrombin time, *CPVB* continuous paravertebral block, *OR* odds ratio

^a^Chi-square test

## Discussion

In our study, there were no cases with severe CPVB-related complications. American Society of Regional Anesthesia (ASRA) guidelines recommend neuraxial blocks should be avoided in patients with known coagulopathy from any cause. Guidelines also state the interval between neuraxial blocks and systemic heparinization should exceed 60 min [10]. On the other hand, Okitsu et al. showed that CPVB-related hematoma did not occur in patients with risk of bleeding who underwent cardiovascular surgery including non-open-heart surgery [16]. If paravertebral hematoma is formed, the incidence of neuropathy seems to be rare [17, 18]. The current report showed that performance of CPVB was possible without severe complications even in cardiac surgery. However, we showed that even mild coagulopathy was associated with CPVB-related bleeding. In our report, although there were no patients with thrombocytopenia, preoperative antiplatelet, or anticoagulant, the dosage of intravenous unfractionated heparin was larger and the interval between CPVB and systemic heparinization was shorter than in the report by Okitsu et al. Even if it is difficult to reduce the dosage of intravenous heparin in open-heart surgery, we probably should confirm that patients’ PT-INR is in normal range and perform CPVB earlier to make it safe.

Although MICS is associated with smaller incision, pain is an important factor that degrades respiratory function and prevents ambulation after thoracic surgery. Although continuous intravenous opioid infusion may be useful and safe for cardiac surgery, it is related to cough suppression and respiratory depression and can prolong duration of intubation, particularly in elderly patients*.* Recent studies reported that the use of very short-acting opioids, such as remifentanil, reduced mechanical ventilation time in patients undergoing cardiac surgery [19–21]. However, Komatsu et al. reported that the use of remifentanil was associated with more episodes of bradycardia and hypotension compared with the use of other short-acting opioids [22]. Although there are advantages of very short-acting opioids, such as remifentanil, we must consider intraoperative hemodynamic deterioration and exacerbation of postoperative pain. Carmona et al. reported that CPVB analgesia was an acceptable safe technique for early extubation with optimal pain control retrospectively compared with intravenous analgesia with opioids in 37 patients who underwent cardiac surgery [23]. Some authors showed that CPVB was a good pain reliever similar to EA in thoracic surgery [12–15], so we hypothesized that CPVB could potentially replace EA as the standard procedure in open-heart surgery. Although we did not record any pain scales to evaluate the degree of pain, our fentanyl consumption was small compared to past reports [19, 24–26], and our patients did not require multiple analgesics as we expected. Surprisingly, postextubation respiratory failure was not observed in any of our patients.

There are several limitations to this report. First, we performed no diagnostic modalities, such as magnetic resonance imaging or computed tomography, so the actual rate of hematoma incidence was unclear. Second, this was a retrospective, non-comparable study and had a small sample size. It was not clear whether CPVB reduced fentanyl consumption and contribute to early extubation. Third, we did not record any scales to evaluate the degree of pain, such as a numeric rating scale or visual analogue scale, so it was difficult to assess the pain relieving efficacy in this study. Randomized controlled trials (RCT) are needed to establish superiority of CPVB in comparison to other analgesic techniques in open-heart surgery.

In conclusion, there were no cases of severe CPVB-related complications or postextubation respiratory failure in any of our patients who underwent MICS. The current report showed that performance of CPVB was possible without severe complications even in cardiac surgery. However, even mild coagulopathy was associated with CPVB-related bleeding in our report, indicating CPVB must be considered carefully, particularly in open-heart surgery.
